# Dynamic Constitutive Model of Ultra-High Molecular Weight Polyethylene (UHMWPE): Considering the Temperature and Strain Rate Effects

**DOI:** 10.3390/polym12071561

**Published:** 2020-07-14

**Authors:** Kebin Zhang, Wenbin Li, Yu Zheng, Wenjin Yao, Changfang Zhao

**Affiliations:** 1 ZNDY of Ministerial Key Laboratory, Nanjing University of Science and Technology, Nanjing 210094, China; kb2018@njust.edu.cn (K.Z.); zhengyu@njust.edu.cn (Y.Z.); njyaowj@163.com (W.Y.); 2 School of Mechanical Engineering, Nanjing University of Science and Technology, Nanjing 210094, China; lackychang@njust.edu.cn

**Keywords:** UHMWPE, strain rate, temperature, constitutive model, SHPB

## Abstract

The temperature and strain rate significantly affect the ballistic performance of UHMWPE, but the deformation of UHMWPE under thermo-mechanical coupling has been rarely studied. To investigate the influences of the temperature and the strain rate on the mechanical properties of UHMWPE, a Split Hopkinson Pressure Bar (SHPB) apparatus was used to conduct uniaxial compression experiments on UHMWPE. The stress–strain curves of UHMWPE were obtained at temperatures of 20–100 °C and strain rates of 1300–4300 s^−1^. Based on the experimental results, the UHMWPE belongs to viscoelastic–plastic material, and a hardening effect occurs once UHMWPE enters the plastic zone. By comparing the stress–strain curves at different temperatures and strain rates, it was found that UHMWPE exhibits strain rate strengthening and temperature softening effects. By modifying the Sherwood–Frost model, a constitutive model was established to describe the dynamic mechanical properties of UHMWPE at different temperatures. The results calculated using the constitutive model were in good agreement with the experimental data. This study provides a reference for the design of UHMWPE as a ballistic-resistant material.

## 1. Introduction

Ultra-high molecular weight polyethylene (UHMWPE) is extensively applied in various fields, such as the aerospace, transportation, and medical fields, due to its low density, non-toxicity, and excellent impact resistance [1,2,3]. In the domain of weapons manufacturing, UHMWPE has great potential for application as a ballistic-resistant material due to its great impact resistance and high specific energy absorption [4]. While studying the ballistic performance of UHMWPE, in addition to exploring the deformation of UHMWPE at high strain rates, the effect of ambient temperature on the mechanical properties of UHMWPE should be considered [5,6,7]. Hence, it is important to study the dynamic mechanical properties of UHMWPE at different temperatures.

To broaden the application of UHMWPE, many studies have been carried out on its mechanical properties. Bergström et al. [8] compared the abilities of “J2-plasticity” theory [9], the “Arruda–Boyce” model [10,11], the “Hasan–Boyce” model [12], and the “Bergström–Boyce” model [13] to reproduce the tensile and compressive mechanical behaviours of UHMWPE under quasi-static conditions. Furthermore, a new hybrid model was proposed based on previous theories to effectively predict the quasi-static tensile and compressive mechanical behaviours of UHMWPE. Kurtz et al. [14] studied the effects of thermal treatment and γ irradiation on the mechanical properties of UHMWPE under static conditions, finding that the mechanical properties of UHMWPE could be significantly changed by varying the mode of thermal treatment and the radiation dose. Furthermore, the thermodynamic behaviours of UHMWPE could be precisely predicted by the Arrhenius model at temperatures of 20–60 °C. By comparing the uniaxial compression experiments of low-density polyethylene and UHMWPE under static and dynamic conditions, Xu et al. [15] found that UHMWPE had a stronger energy absorption capacity than low-density polyethylene under the same conditions. Qin et al. [16] conducted quasi-static experiments to study the uniaxial compression mechanical properties of UHMWPE with a molecular weight of 2.25–5.09 million, the results of which showed that the yield stress of UHMWPE increased first and then decreased with the increase in the molecular weight. Zhang et al. [17] explored the deformational behaviours of UHMWPE at strain rates of 0.001–3300 s^−1^ and established a constitutive model for UHMWPE in the dynamic plastic stage. By studying the effect of hybrid braided UHMWPE fibres on the impact properties and residual bending stiffness of CFRPs through falling weight impact tests, four-point bending tests, and finite element analysis, Hu et al. [18] explained the mechanism for restricting damage propagation in impact tests and improved the damage tolerance and integrity of the structure. However, the elastic-plastic constitutive model considering both strain rate effect and temperature effect was not studied in Ref. [8,9,10,11,12,13,14,15,16,17,18]. On this basis, the research considering the strain rate effect and temperature effect was carried out in the present paper.

The split Hopkinson pressure bar (SHPB) is a good tool to study the mechanical behavior of materials at the strain rate range of 10^2^–10^4^. In this paper, the uniaxial compression mechanical properties of UHMWPE at temperatures of 20–100 °C and strain rates of 1300–4300 s^−1^ were studied by SHPB. In this experiment, the temperature range included the maximum storage temperature of the projectile of 50 °C and the maximum working temperature of the projectile of 70 °C, with the strain rate range being close to that of projectile impact [15]. To understand the dynamic failure behaviours of UHMWPE from the perspective of the molecular structure, after the SHPB experiment, the UHMWPE samples were observed and analysed using an FEI Quanta 250F field-emission environmental scanning electron microscope. The UHMWPE stress–strain curves obtained from the experiments were used to modify the Sherwood–Frost constitutive model, and the modified constitutive model was used to describe the dynamic mechanical properties of UHMWPE at different temperatures.

## 2. Materials and Methods

### 2.1. Materials and Sample Preparation

**Figure 1:**
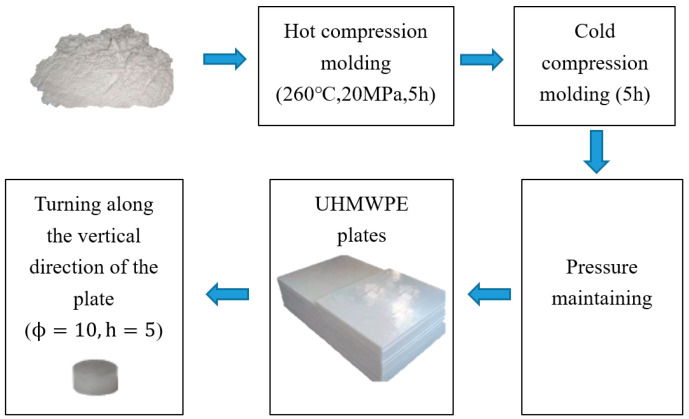
The manufacturing process of UHMWPE specimens.

In this study, commercially produced moulded UHMWPE, which is widely used in aircraft, automobiles, and medical equipment, was utilized. The moulding plate of UHMWPE was obtained from the moulding machine. Figure 1 shows the manufacturing process of the samples. The UHMWPE powder was pressed in a hot press at a moulding temperature of 260 °C and a pressure of 20 MPa. After 5 h of hot pressing followed by 5 h of cold pressing in a cold press, the UHMWPE powder was cooled and shaped to obtain 1000 mm × 1000 mm × 20 mm UHMWPE sheets. Table 1 shows some basic performance parameters of the UHMWPE material provided by the manufacturer. The UHMWPE dynamic compression specimens were designed by referencing Ref. [19]. The specimens used in the SHPB experiments were cylinders with 10-mm diameters and 5-mm heights, which were obtained by turning the UHMWPE sheets. Xu et al. [15] proved that there is almost no difference in the tensile mechanical properties of UHMWPE in different material directions, which meant UHMWPE can be considered as isotropic material. Therefore, we also believe that the UHMWPE samples used in this paper are isotropic. To the convenience of machining, the axes of all the cylindrical compression specimens prepared in this experiment were perpendicular to the surface of the UHMWPE sheet. The prepared compression specimens were stored at room temperature for 48 h, and the residual stress was eliminated before the experiment [17].

### 2.2. SHPB Testing

**Figure 2:**
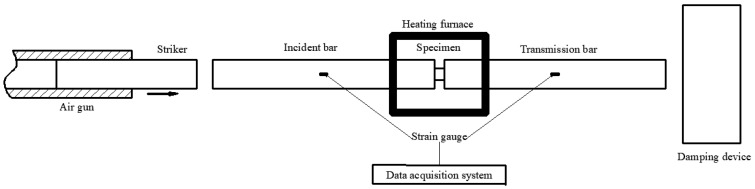
Schematic diagram of the SHPB experiment.

In the present study, the SHPB experimental apparatus was used to explore the dynamic mechanical properties of UHMWPE. Figure 2 shows the schematic diagram of the SHPB apparatus. The SHPB apparatus mainly consisted of an air gun, bullets, an incident bar, a transmission bar, and a damping system. Since UHMWPE is a low-impedance material, all the bars used in this experiment were made of 7A04 aluminium alloy to obtain a relatively high signal-to-noise ratio (SNR) [20]. Table 2 shows the detailed parameters of the SHPB used in this experiment.

Prior to the experiment, the UHMWPE specimen was sandwiched between the incident bar and the transmission bar. The bullets were made to hit the incident bar at different speeds by controlling the gas pressure in the air gun. The bullet impact caused an incident stress pulse $\varepsilon_{i}\left(t\right)\;$ in the incident bar. As the stress pulse reached the specimen, the specimen was deformed under the stress pulse, a backward reflection pulse $\varepsilon_{r}\left(t\right)$ was generated in the incident bar, and a forward transmission pulse $\varepsilon_{t}\left(t\right)$ was produced in the transmission bar. These pulse signals were measured by strain gauges glued to the incident and transmission bars. The true stress $\sigma_{T}$, true strain $\varepsilon_{T}$, and strain rate $\dot{\varepsilon }$ of UHMWPE were calculated using the following equations [21,22]:

(1) $$\dot{\varepsilon }=-\frac{2C_{0}}{L_{S}}\varepsilon_{r}\left(t\right)$$

(2) $$\varepsilon_{E}=\int_{0}^{t}\dot{\varepsilon }dt$$

(3) $$\sigma_{E}=\frac{A_{0}E_{0}}{A_{S}}\varepsilon_{t}\left(t\right)$$

(4) $$\sigma_{T}=\sigma_{E}\left(1-\varepsilon_{E}\right)$$

(5) $$\varepsilon_{T}=-\ln\left(1-\varepsilon_{E}\right)$$

where $A_{S}$ and $L_{S}$ are the initial cross-sectional area and length of the specimen, respectively, and *C*_0_, *A*_0_, and *E*_0_ denote the elastic wave velocity, cross-sectional area, and elastic modulus of the bar, respectively. Table 3 shows the pressure of the air gun and the strain rates of the UHMWPE specimens in the experiments.

To study the dynamic mechanical properties of UHMWPE at different temperatures, the specimen was heated to the specified temperature and maintained for 5 min before the SHPB experiment was carried out [23]. The temperatures in the experiment were 20, 50, 70, and 100 °C. Before the experiment, the effect of the temperature on the SHPB was eliminated by an empty bar experiment. To improve the accuracy of the experimental data, experiments under the same working conditions were repeated three times to obtain the average value.

## 3. Results and Discussion

### 3.1. Strain Rate Effect

**Figure 3:**
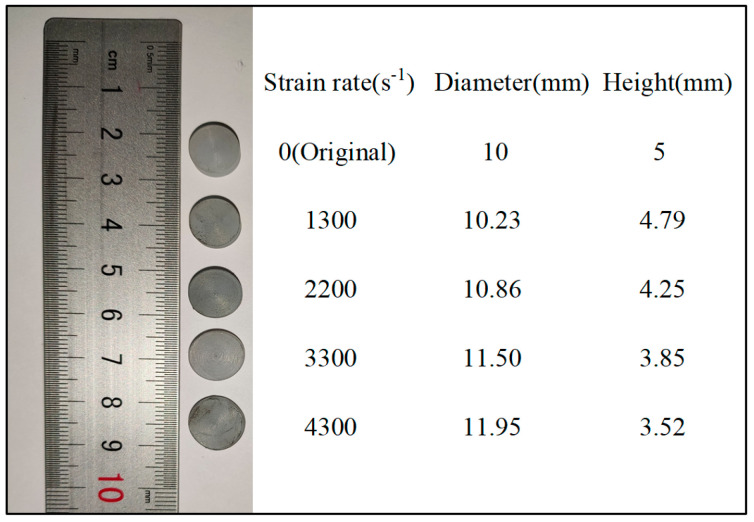
UHMWPE specimens before and after the SHPB experiment.

To study the strain rate effect of the UHMWPE, SHPB experiments were conducted at 20 °C. Figure 3 shows the deformation of the UHMWPE specimen before and after the SHPB test. As the strain rate increased, the thickness of the specimen decreased gradually, the diameter increased gradually, and the deformation became more significant after the experiment.

**Figure 4:**
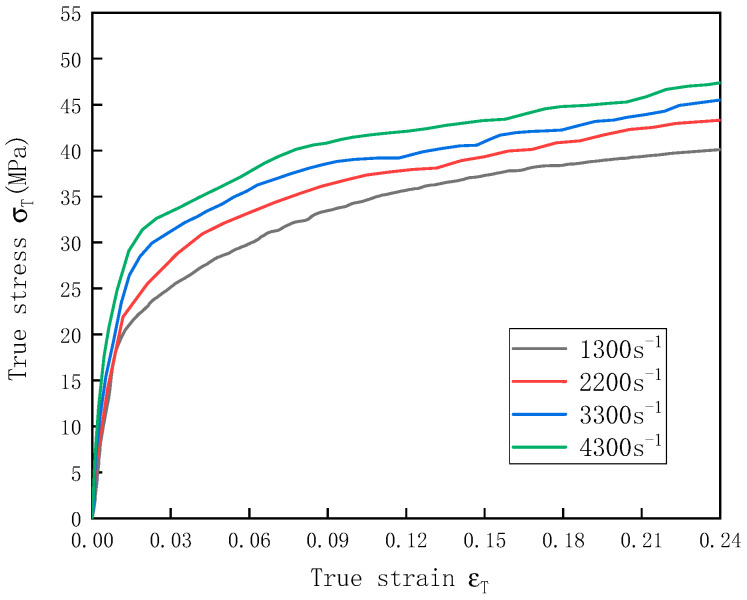
Stress–strain curves of UHMWPE at a temperature of 20 °C and strain rates of 1300–4300 s^−1^.

The stress–strain curves of the UHMWPE at 20 °C and strain rates of 1300–4300 s^−1^ were obtained (Figure 4). Each of the dynamic compression stress–strain curves increased linearly in the beginning and entered a yield stage with the increase in the load. After the yield stage, the stress grew slowly but the strain continued to rise, showing significant plastic deformation. By comparing the dynamic UHMWPE stress–strain curves at different strain rates, it was found that the compressive stress exhibited a significant strain rate effect. The elastic modulus and yield stress of the UHMWPE increased as the strain rate increased, which were shown in Table 4.

The yield stress of UHMWPE was calculated by using the inverse method [24]. Like the method in the study of Qin et al. [16], the secant modulus was calculated by the two points of strain equal to 0.05% and 0.25%, which was used as the elastic modulus of UHMWPE. The formula is as follows:

(6) $$E=\frac{\sigma_{0.25\%}-\sigma_{0.05\%}}{\varepsilon_{0.25\%}-\varepsilon_{0.05\%}}$$

where E is the elastic modulus of UHMWPE, $\sigma_{0.25\%}$ and $\sigma_{0.05\%}$ are the stress value when the strain reaches 0.25% and 0.05%, respectively, and $\varepsilon_{0.25\%}$ and $\varepsilon_{0.05\%}$ are the strain value when the strain reaches 0.25% and 0.05%, respectively.

### 3.2. Temperature Effect

**Figure 5:**
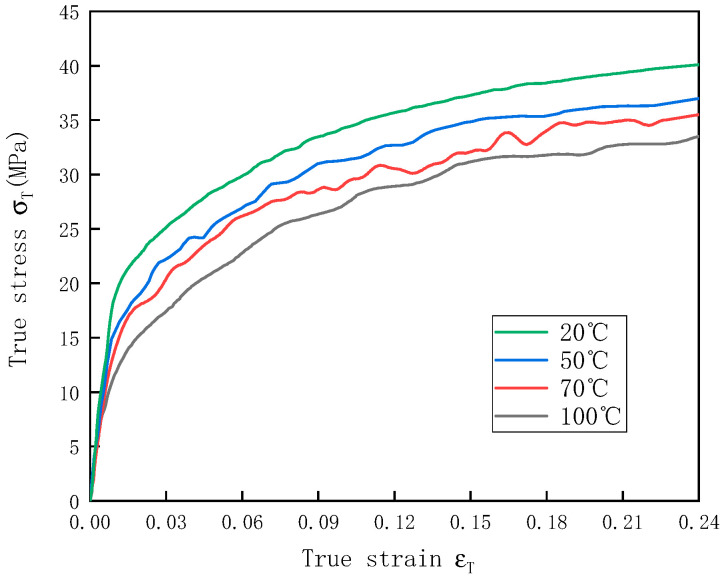
Stress–strain curves of UHMWPE at temperatures of 20–100 °C and a strain rate of 1300 s^−1^.

UHMWPE is a high-molecular-weight polymer, and the effects of temperature on the mechanical properties of the UHMWPE are of great concern. Figure 5 shows the stress–strain curves of the UHMWPE at temperatures of 20–100 °C and a strain rate of 1300 s^−1^. By comparing the dynamic UHMWPE stress–strain curves at different temperatures, it was found that the compressive stress was characterized by a significant temperature effect. The elastic modulus and yield stress of UHMWPE decreased as the temperature decreased (Table 4). This indicated that with the increase in the temperature, the UHMWPE chain became softer and was more likely to deform at the same impact velocity, resulting in decreases in the yield stress and elastic modulus [25,26].

### 3.3. Microscopic Deformation Behaviour

**Figure 6:**
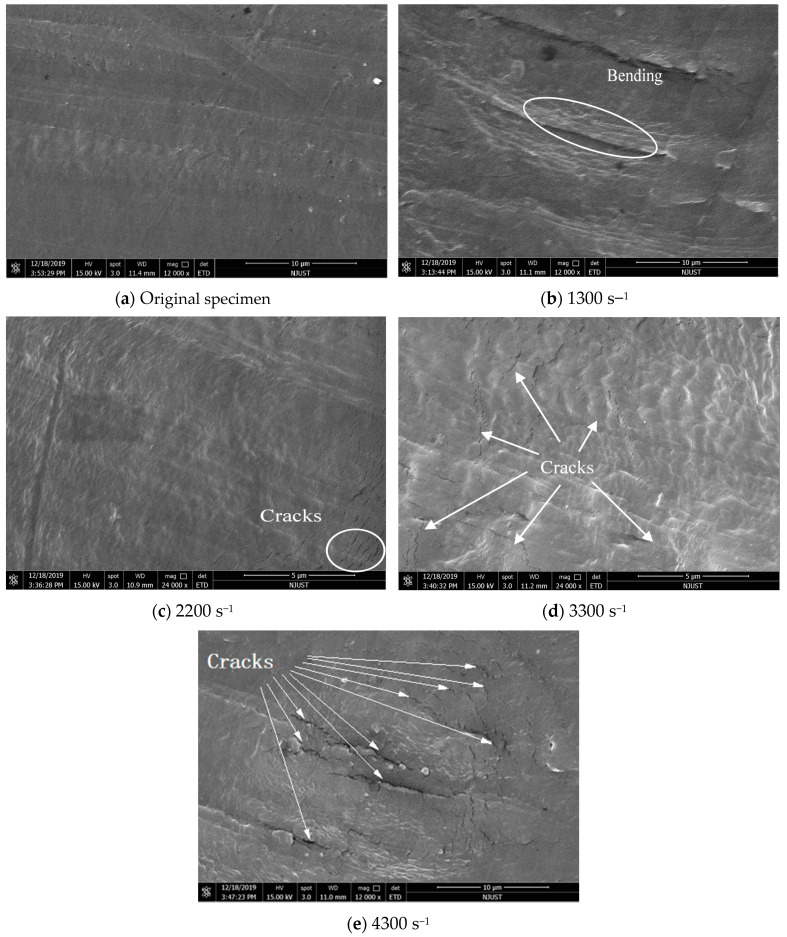
SEM images of UHMWPE specimens before and after the SHPB experiments.

To more clearly understand the dynamic deformation behaviour of UHMWPE microscopically, the specimens before and after SHPB test were cut along the axial direction and then sprayed with gold. The deformations of the cross-sections of the UHMWPE specimens under the impact of bullets with different velocities were observed using a FEI Quanta 250F field-emission environmental scanning electron microscope. Figure 6 shows the results.

The UHMWPE chains were orderly arranged before the SHPB experiment, and the UHMWPE chains were bent at a compressive strain rate of 1300 s^−1^. As the strain rate increased, cracks were found in the UHMWPE chains, and with the further increase in the strain rate, cracks propagated and grew so that bulging appeared in the UHMWPE chains between cracks. As the strain rate reached 4300 s^−1^, this phenomenon became so obvious that the UHMWPE chains could not be identified. This implied that with the increase in the strain rate, the molecular mobility ratio of the UHMWPE chains decreased, and the molecular chains hardened, resulting in crack failure [27,28].

As observed by the electron microscope, the microscopic morphologies of the UHMWPE specimens at a strain rate of 1300 s^−1^ and temperatures of 20–100 °C were not significantly different from that at room temperature. Thus, the microscopic morphology of the UHMWPE at other temperatures is not discussed in this paper.

## 4. Constitutive Model

Through investigation, the constitutive models that can describe the dynamic mechanical properties of polymers have ZWT [29] constitutive model, overstress constitutive model [30], and Sherwood-Frost constitutive model [31]. The ZWT constitutive model can describe well the mechanical properties of polymer materials in the strain rate range of 10^−4^–10^3^ s^−1^, but it cannot accurately describe the viscoplastic mechanical properties after yielding, and it can only describe the mechanical properties in the 8% deformation range. The overstress model can describe the elastic and plastic mechanical properties in stages, but it does not consider the effect of temperature. The Sherwood-Frost constitutive model includes a temperature term and a strain rate term, which can describe well the mechanical properties of polymers at different temperatures and strain rates.

Based on the SHPB experiment, the UHMWPE was characterized by apparent strain rate strengthening and temperature softening effects. The stress-strain behavior of UHMWPE was similar to that of the polyurethane foam studied by Frost [31,32]. In this study, by modifying the Sherwood–Frost constitutive model proposed by Frost, a constitutive equation was obtained to describe the dynamic uniaxial compression properties of UHMWPE.

The Sherwood–Frost constitutive equation includes a shape function f(ε), a temperature function H(T), a density function G(ρ), and a strain rate function $M\left(\varepsilon ,\dot{\varepsilon }\right)$, as follows:

(7) $$\sigma_{c}=H\left(T\right)G\left(\rho \right)M\left(\varepsilon ,\dot{\varepsilon }\right)f\left(\varepsilon \right)$$

The density range of UHMWPE on the market is between 0.93–0.98 g/cm^3^, so the change of density is very small, which results in a small change of G(ρ). Hence, the influence of the density term on the entire constitutive model can be ignored, and Equation (7) is simplified to the following:

(8) $$\sigma_{c}=H\left(T\right)M\left(\varepsilon ,\dot{\varepsilon }\right)f\left(\varepsilon \right)$$

where f(ε) is a polynomial function that describes the shape of the stress–strain curve:

(9) $$f\left(\varepsilon \right)=\overset{10}{\underset{n=1}{\sum }}A_{n}\varepsilon^{n}$$

Nagy et al. proposed the use of an exponential strain rate [33], and thus, $M\left(\varepsilon ,\dot{\varepsilon }\right)$ was expressed as follows:

(10) $$M\left(\varepsilon ,\dot{\varepsilon }\right)={\left(\dot{\varepsilon }/{\dot{\varepsilon }}_{0}\right)}^{n\left(\varepsilon \right)}$$

(11) $$n\left(\varepsilon \right)=b_{1}+b_{2}\varepsilon$$

The temperature function was expressed as follows:

(12) $$H\left(T\right)=\frac{\sigma_{c}}{M\left(\varepsilon ,\dot{\varepsilon }\right)f\left(\varepsilon \right)}$$

where $A_{n}$ is a parameter that describes the stress–strain shape of the material, which was determined by fitting with a tenth-order polynomial, ${\dot{\varepsilon }}_{0}$ denotes the lowest possible strain rate in the experiment, $b_{1}$ and $b_{2}$ are parameters of the material determined by the experiment, and H(T)=1 at room temperature.

### 4.1. Establishment of Constitutive Model

#### 4.1.1. Strain Rate Effect

First, the shape function f(ε) in the constitutive equation was fitted. f(ε) was determined by an SHPB compression test at the lowest possible strain rate at room temperature. In this experiment, the room temperature was 20 °C, and the lowest possible strain rate was ${\dot{\varepsilon }}_{0}$ = 1300 s^−1^. By fitting the stress–strain curve of the UHMWPE under these conditions, parameter $A_{n}$ was obtained and is listed in Table 5.

According to Equations (10) and (12), $M\left(\varepsilon ,\dot{\varepsilon }\right)=H\left(T\right)=1$, so the stress–strain relationship of UHMWPE was reduced to the shape function f(ε). Therefore, Equation (8) becomes the following:

(13) $$\sigma_{c}=1f\left(\varepsilon \right)$$

Due to the inherent rate dependence of the polymer matrix and aerodynamic damping [28], the UHMWPE was strain rate dependent. To explore the effect of the strain rate function $M\left(\varepsilon ,\dot{\varepsilon }\right)\;$ on the mechanical properties of UHMWPE at room temperature, Equation (8) was changed to the following:

(14) $$M\left(\varepsilon ,\dot{\varepsilon }\right)=\frac{\sigma_{c}}{f\left(\varepsilon \right)}=\frac{\sigma_{c}}{{\left[\sigma_{c}\right]}_{1300s^{-1}}}$$

**Figure 7:**
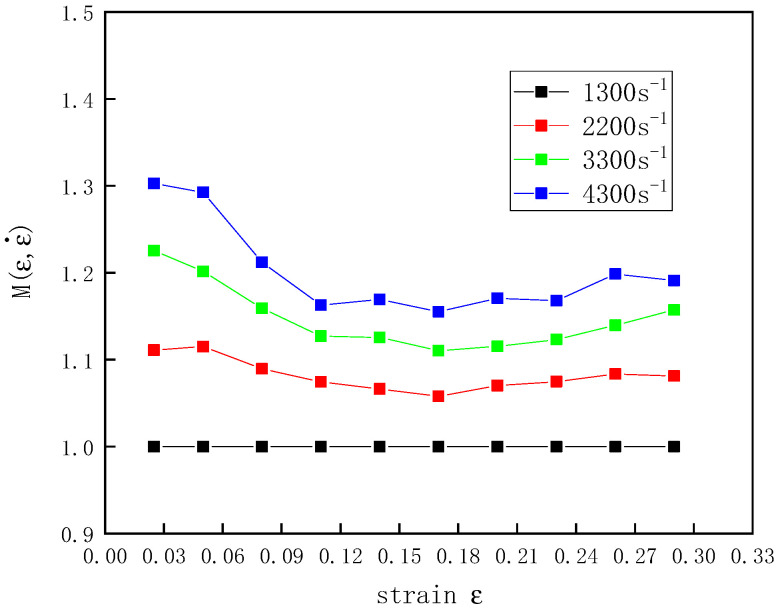
The relationship between $M\left(\varepsilon ,\dot{\varepsilon }\right)$ and ε at a temperature of 20 °C and different strain rates.

Based on the stress–strain curves at strain rates of 1300–4300 s^−1^ at room temperature, the relationship between $\;M\left(\varepsilon ,\dot{\varepsilon }\right)$ and the strain ε was obtained (Figure 7).

To obtain the parameters in n(ε), the logarithm of Equation (10) was taken to obtain the following:

(15) $$n\left(\varepsilon \right)=\frac{\ln M\left(\varepsilon ,\dot{\varepsilon }\right)}{\ln\dot{\varepsilon }/{\dot{\varepsilon }}_{0}}$$

**Figure 8:**
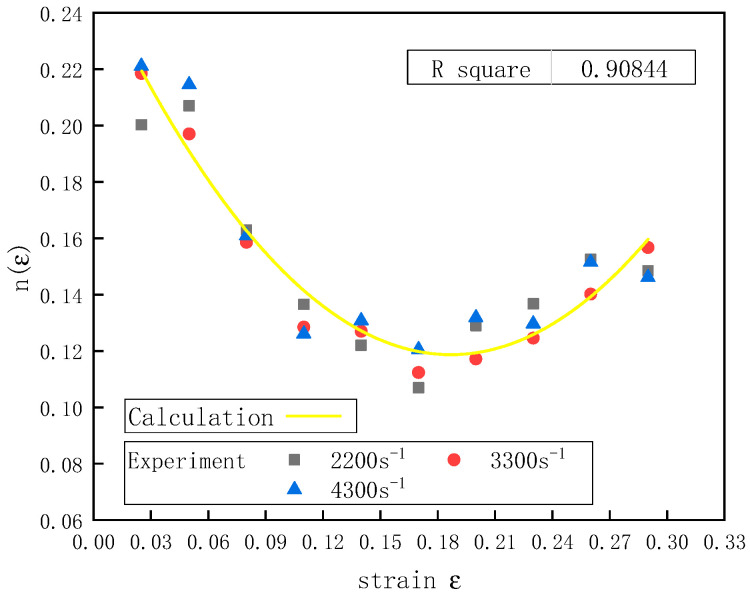
The relationship between n(ε)  and ε at a temperature of 20 °C and different strain rates.

Figure 8 shows the relationship between n(ε)  and the strain *ε*. There was a non-linear relationship between the strain rate index function n(ε) and the strain ε of the UHMWPE, so a quadratic function $b_{3}\varepsilon^{2}$ was added to Equation (11):

(16) $$n\left(\varepsilon \right)=b_{1}+b_{2}\varepsilon +b_{3}\varepsilon^{2}$$

Equation (16) was fitted by the data in Figure 8, and the fitted curve correlated well with the experimental results. Table 6 shows the values of parameters $b_{1}$, $b_{2}$, and $b_{3}$.

.

**Figure 9:**
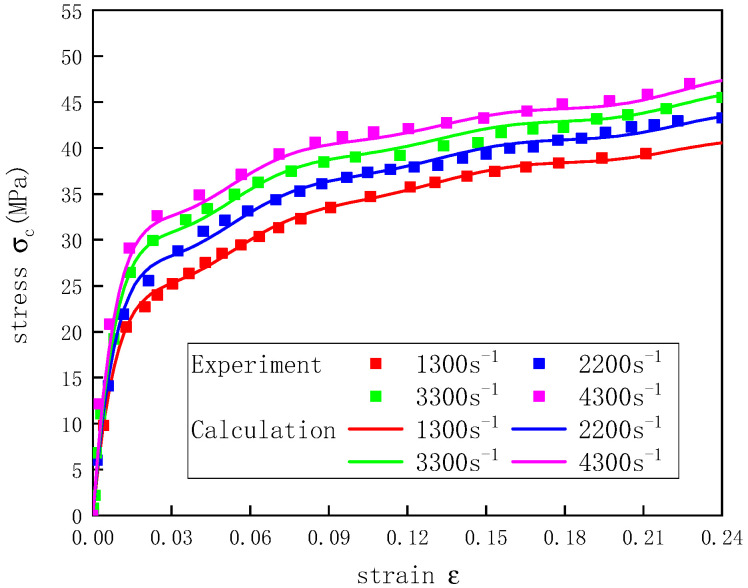
Stress–strain curves of UHMWPE at a temperature of 20 °C and different strain rates.

Based on the parameters of the shape function  f(ε)  and the strain rate function $M\left(\varepsilon ,\dot{\varepsilon }\right)$ obtained through fitting, the stress–strain curves of UHMWPE at a temperature of 20 °C and strain rates of 1300–4300 s^−1^ were calculated and compared with the experimental values (Figure 9). The calculated curves were in good agreement with the experimental data, which showed that the dynamic UHMWPE stress–strain curves at different strain rates could be described well by modifying the n(ε) function in the Sherwood–Frost constitutive equation.

#### 4.1.2. Temperature Effect

The experiments showed that the temperature and strain rate had similar effects on the mechanical properties of the UHMWPE. As the temperature increased and the strain rate decreased, the stress–strain curves of the UHMWPE varied similarly. The stress of the UHMWPE varied with the increase in the strain and the temperature. Hence, in this study, the temperature function H(T) of the constitutive equation was described in a form similar to that of the strain rate function $M\left(\varepsilon ,\dot{\varepsilon }\right)$. The temperature function H(ε,T) was proposed with reference to the strain rate function:

(17) $$H\left(\varepsilon ,T\right)={\left(T_{0}/T\right)}^{m\left(\varepsilon \right)}$$

where $T_{0}$ is the room temperature in the experiment, $T_{0}=20\;{}^{\circ}C$, and m(ε) is a function of the strain. In this study, the stress-strain curve of UHMWPE at 20~100 °C and the strain rate of 1300 s^−1^ were used to study the temperature function H(ε,T). Here $M\left(\varepsilon ,\dot{\varepsilon }\right)=1$, Equation (8) became the following:

(18) $$\sigma_{c}=H\left(\varepsilon ,T\right)f\left(\varepsilon \right)$$

**Figure 10:**
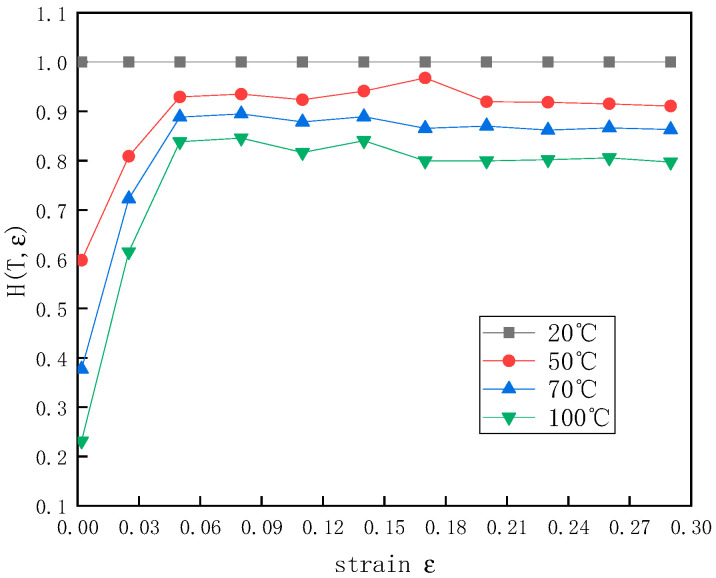
The relationship between H(ε,T)  and ε at a strain rate of 1300 s^−1^ and different temperatures.

The relationship between the temperature function H(ε,T) and the strain ε was as follows, the results of which are shown in Figure 10:

(19) $$H\left(\varepsilon ,T\right)=\frac{\sigma_{c}}{f\left(\varepsilon \right)}=\frac{\sigma_{c}}{{\left[\sigma_{c}\right]}_{20{}^{\circ}C}}$$

To study the relationship between the strain function m(ε) and strain in the temperature function H(ε,T), the logarithm of Equation (17) was taken to obtain the following:

(20) $$m\left(\varepsilon \right)=\frac{\ln H\left(T\right)}{\ln T_{0}/T}$$

**Figure 11:**
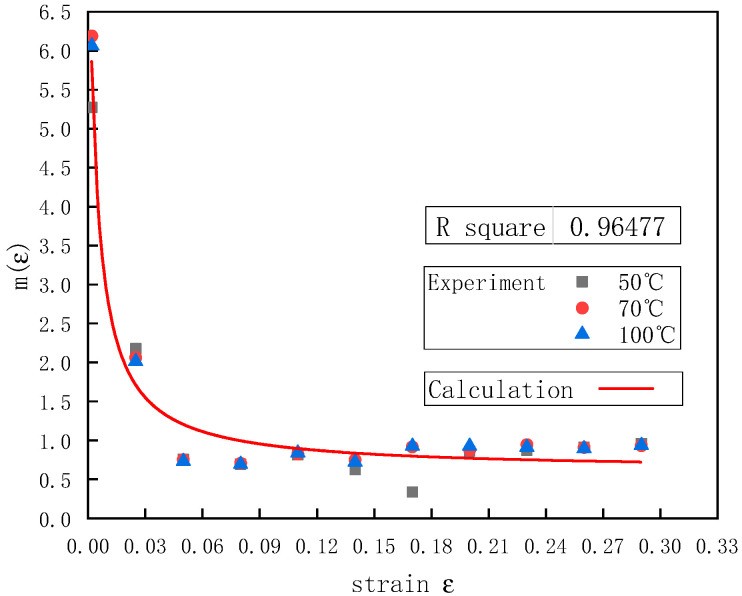
The relationship between m(ε)  and ε at a strain rate of 1300 s^−1^ and different temperatures.

Based on the data in Figure 10, the relationship between m(ε) and the strain ε was obtained (Figure 11).

The strain function m(ε) in the temperature function was described well by the following function:

(21) $$m\left(\varepsilon \right)=\frac{c_{2}}{\varepsilon +c_{1}}+c_{3}$$

where $c_{1}$, $c_{2}$, and $c_{3}$ are the material parameters determined by the experiment. Based on the experimental data in Figure 11, Equation (21) was fitted to obtain the values of parameters $c_{1}$, $c_{2}$, and $c_{3}$ (Table 7). Figure 11 shows the comparison of the calculated curves and the experimental results, indicating that there was a good correlation between the calculated curves and the experimental results.

**Figure 12:**
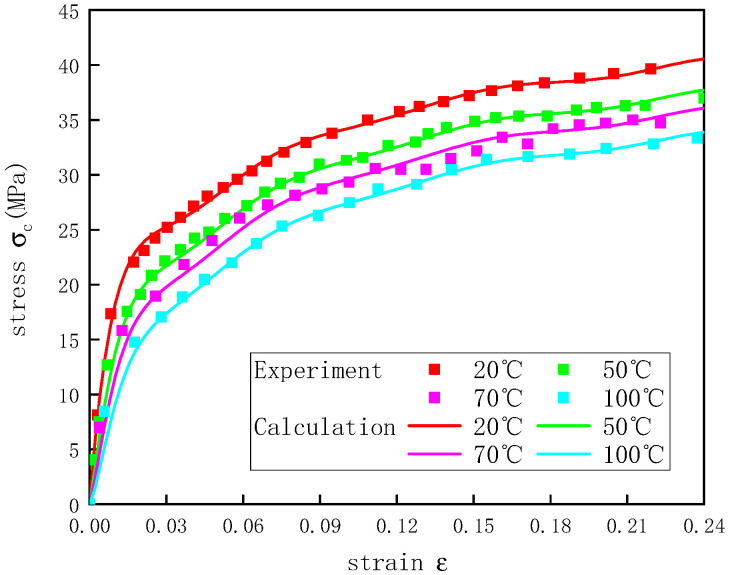
Stress–strain curves of UHMWPE at a strain rate of 1300 s^−1^ and different temperatures.

The stress–strain curves of the UHMWPE at temperatures of 20–100 °C and a strain rate of 1300 s^−1^ were calculated based on the parameters of the shape function f(ε)  and the temperature-dependent function H(ε,T), which were obtained by fitting (Figure 12). The calculated curves were in good agreement with the experimental results, indicating that the H(ε,T) function proposed was able to describe the stress–strain curves of the UHMWPE well at a strain rate of 1300 s^−1^ and different temperatures.

In summary, by modifying the strain rate function and adding the temperature function proposed by the strain rate function for reference, the following constitutive equation describing UHMWPE was obtained:

(22) $$\{\begin{array}{c} \sigma_{c}=H\left(\varepsilon ,T\right)M\left(\varepsilon ,\dot{\varepsilon }\right)f\left(\varepsilon \right)\\ f\left(\varepsilon \right)=\sum_{n=1}^{10}A_{n}\varepsilon^{n}\\ M\left(\varepsilon ,\dot{\varepsilon }\right)={\left(\dot{\varepsilon }/{\dot{\varepsilon }}_{0}\right)}^{b_{1}+b_{2}\varepsilon +b_{3}\varepsilon^{2}}\\ H\left(\varepsilon ,T\right)={\left(T_{0}/T\right)}^{\frac{c_{2}}{\varepsilon +c_{1}}+c_{3}} \end{array}\;$$

The parameter values in this equation are shown in Table 5, Table 6 and Table 7.

### 4.2. Verification of Constitutive Equation

**Figure 13:**
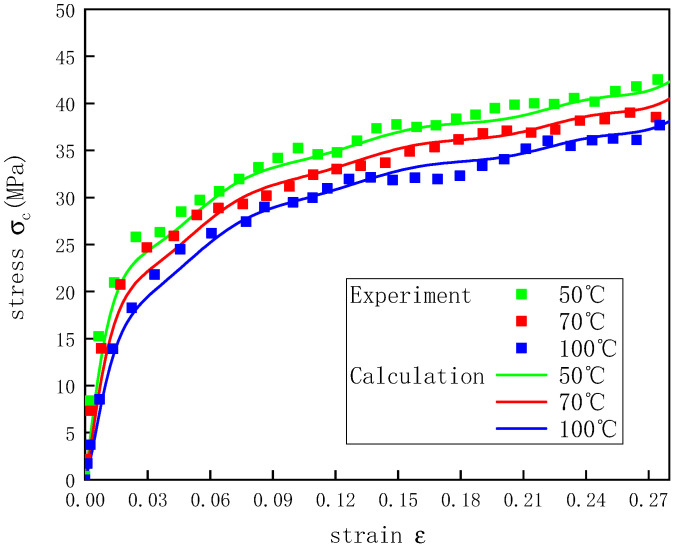
Stress–strain curves of UHMWPE at a strain rate of 2200 s^−1^ and different temperatures.

**Figure 14:**
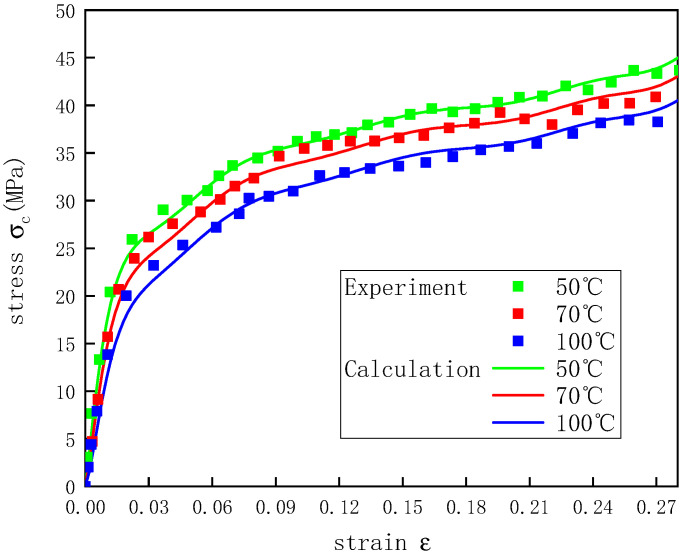
Stress–strain curves of UHMWPE at a strain rate of 3300 s^−1^ and different temperatures.

**Figure 15:**
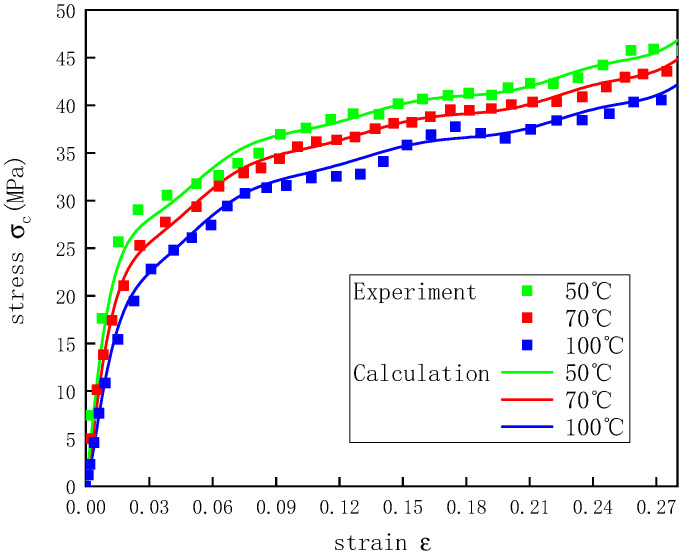
Stress–strain curves of UHMWPE at a strain rate of 4300 s^−1^ and different temperatures.

To verify that the constitutive model proposed in this study could better describe the stress–strain curves of the UHMWPE at different temperatures under dynamic conditions, nine combinations of temperatures and strain rates were selected to verify Equation (22). Figure 13, Figure 14 and Figure 15 show how the actual and predicted stress responses of the UHMWPE varied with the strain at strain rates of 2200, 3300, and 4300 s^−1^. For the given experimental data, this model could predict well the experimental results of the UHMWPE in the plastic stage, and there were some errors in the elastic stage, but all the errors were less than 7%. The errors during the elastic stage likely occurred because it was difficult for the UHMWPE to achieve a constant strain rate in the elastic stage of the material during the SHPB experiments [34].

## 5. Conclusions

SHPB experiments were carried out to study the uniaxial compression properties of UHMWPE under dynamic conditions at different temperatures. By comparing the stress–strain curves of UHMWPE at different temperatures under dynamic conditions, it was found that the yield stress and the elastic modulus of the UHMWPE increased as the strain rate was increased and decreased as the temperature was increased. The UHMWPE exhibited strain rate strengthening and temperature softening effects.

The microscopic deformation behaviours of the UHMWPE specimens after dynamic compression were observed and analysed using a FEI Quanta 250F field-emission environmental scanning electron microscope. As the strain rate increased, the molecular mobility ratio of the UHMWPE chains decreased, and the molecular chains hardened, resulting in crack failure.

The Sherwood–Frost constitutive model was used to fit the stress–strain curves of the UHMWPE under different compressive strain rates, the results of which showed that there was a non-linear relationship between the strain rate index function n(ε) and strain ε of the UHMWPE. The strain rate effect of the UHMWPE under dynamic uniaxial compression could be effectively described by introducing the quadratic function $b_{3}\varepsilon^{2}$.

The experiments showed that the temperature and the strain rate had similar effects on the mechanical properties of the UHMWPE. With reference to the strain rate function $M\left(\varepsilon ,\dot{\varepsilon }\right)\;$ in the Sherwood–Frost constitutive equation, a temperature function H(ε,T) was proposed. Based on the experimental results, H(ε,T) could describe the temperature effect of the uniaxial compression properties of UHMWPE well under dynamic conditions.

The constitutive model obtained by modifying the Sherwood–Frost model could effectively describe the stress–strain curves of UHMWPE at temperatures of 20–100 °C and strain rates of 1300–4300 s^−1^, which not only provides a data reference and theoretical support for the design of UHMWPE as a ballistic-resistant material but also serves as a reference for the secondary development of a UHMWPE dynamic compression constitutive model for numerical simulations.

We have started the next research work, that is, studying the tensile fracture failure of UHMWPE under dynamic conditions, for the purpose of predicting the perforation characteristics of UHMWPE in ballistic impact tests.

## Figures and Tables

**Table 1:** Properties parameters of UHMWPE.

Density ρ (g/cm^3^)	Elastic Modulus E (MPa)	Poisson’s Ratio ν	Melting Point (°C)	Molecular Weight (g/mol)
0.98	450.49	0.46	136	3 million

**Table 2:** Detailed parameters of SHPB.

Elastic Modulus E_0_ (GPa)	Elastic Wave Velocity C_0_ (m/s)	Bar Diameter (mm)	Striker Length (mm)	Incident Bar Length (mm)	Transmission Bar Length (mm)
70	4991	14.5	400	1500	2000

**Table 3:** Loading pressures and strain rates of specimens.

Air Gun Pressure (MPa)	Average Strain Rate (s^−1^)	Maximum Deviation (s^−1^)
0.015	1300	± 50
0.05	2200	± 50
0.1	3300	± 60
0.15	4300	± 90

**Table 4:** Compressive properties of UHMWPE.

Average Strain Rate (s^−1^)	Temperature (°C)	Modulus of Elasticity (MPa)	Yield Stress (MPa)
1300	20	1697.67 ± 32.39	23.99 ± 0.13
2200	20	1908.86 ± 28.36	26.51 ± 0.27
3300	20	2476.46 ± 48.72	29.90 ± 0.46
4300	20	2628.82 ± 23.97	32.62 ± 0.74
1300	50	1455.06 ± 38.81	20.84 ± 0.25
1300	70	1214.88 ± 33.76	18.74 ± 0.32
1300	100	896.86 ± 25.47	16.48 ± 0.24

**Table 5:** Coefficient value $A_{n}$ (MPa) of the shape function f(ε).

*n*	1	2	3	4	5
$A_{n}$	3000.89	−155,721.90	4.45534 × 10^6^	−7.46181 × 10^7^	7.75704 × 10^8^
***n***	**6**	**7**	**8**	**9**	**10**
$A_{n}$	−5.15158 × 10^9^	2.18663 × 10^10^	−5.73634 × 10^10^	8.46851 × 10^10^	−5.37847 × 10^10^

**Table 6:** Experimental constants in n(ε).

$b_{n}$	$b_{1}$	$b_{2}$	$b_{3}$
Value	0.25287	−1.43586	3.8433

**Table 7:** Experimental constants in m(ε).

$c_{n}$	$c_{1}$	$c_{2}$	$c_{3}$
Value	0.00411	0.03198	0.61374

## References

[1] Somg H.J. (2020). Research progress of UHMWPE artificial joint modified by nano materials. Plast. Technol..

[2] He J.M., Xue P., He Y.D. (1996). Properties and applications of UHMWPE. Eng. Plast. Appl..

[3] Chen Z., Wang J.X., Qin D.T. (2001). Properties and application of UHMWPE in machinery. Mech. Eng. Mater..

[4] Huang A.P., Zhu B.C., Jia J.J., Liu Y.Q. (2012). Development and application of UHMWPE. Polym. Bull..

[5] Gao H., Yang H.W. (2014). Study on the ballistic performance of UHMWPE fiber composite target. J. Armored Force Eng. Coll..

[6] Li W., Li J., Ye Y. (2012). Numerical analysis of bullet proof performance of UHMWPE fiber laminate. Weapon Mater. Sci. Eng..

[7] Zhang Y.M. (2018). Ballistic Performance of SiC Ceramic/UHMWPE Composite Armor. Master’s Thesis.

[8] Bergström J.S., Kurtz S.M., Rimnac C.M., Edidin A.A. (2002). Constitutive modeling of ultra-high molecular weight polyethylene under large-deformation and cyclic loading conditions. Biomaterials.

[9] Kurtz S.M., Pruitt L., Jewett C.W., Crawford R.P., Crane D.J., Edidin A.A. (1998). The yielding, plastic flow, and fracture behavior of ultra-high molecular weight polyethylene used in total joint replacements. Biomaterials.

[10] Cioroianu A.R., Spiesz E.M., Storm C. (2013). An improved non-affine arruda-boyce type constitutive model for collagen networks. Biophys. J..

[11] Hossain M., Steinmann P. (2011). Modelling and simulation of the curing process of polymers by a modified formulation of the Arruda-Boyce model. Arch. Mech..

[12] Cho H., Rinaldi R.G., Boyce M.C. (2013). Constitutive modeling of the rate-dependent resilient and dissipative large deformation behavior of a segmented copolymer polyurea. Soft Matter.

[13] Dal H., Kaliske M. (2009). Bergström–Boyce model for nonlinear finite rubber viscoelasticity: Theoretical aspects and algorithmic treatment for the FE method. Comput. Mech..

[14] Kurtz S.M., Villarraga M.L., Herr M.P., Bergström J.S., Rimnac C.M., Edidin A.A. (2002). Thermomechanical behavior of virgin and highly crosslinked ultra-high molecular weight polyethylene used in total joint replacements. Biomaterials.

[15] Xu M.M., Huang G.Y., Feng S.S., Mcshane G., Stronge W. (2016). Static and dynamic properties of semi-crystalline polyethylene. Polymers.

[16] Qin X.P., Zeng C., Wu S.C., Yin X. (2017). Compressibility experiments of UHMWPE with different molecular weights. Chem. Eng. Equip..

[17] Zhang K.B., Li W.B., Wang X.M., Yao W.J., Song P., Zhao C.F. (2020). A constitutive model of the compressive mechanical properties of ultra high molecular weight polyethylene (UHMWPE) at different temperatures and different strain rates. Mater. Res. Express.

[18] Hu Y., Shi Y., Liu D., Guo J., Zhang J., Chen Z. (2020). Damage tolerance of 2-dimentional UHMWPE/CF hybrid woven laminates subjected to low-velocity impact. Mater. Des..

[19] Sun S.F., Wu X.T., Li H.P., Meng Y.P. (2008). Numerical simulation of sample shape and size effect in SHPB experiment. J. Hefei Univ. Technol..

[20] Hughes F., Prudom A., Swallowe G. (2013). The high strain-rate behaviour of three molecular weights ofpolyethylene examined with a magnesium alloy split-Hopkinson pressure bar. Polym. Test..

[21] Yao X.H., Ren H.L., Lin R., Zhang X.Q. (2012). Study on dynamic mechanical properties and energy absorption of polymeric foams. Chin. J. High Press. Phys..

[22] Zou X.T. (2019). Dynamic Mechanical Properties of TB6 Titanium Alloy and Impact Resistance of Its Typical Structure. Master’s Thesis.

[23] Richeton J., Ahzi S., Daridon L., Rémond Y. (2005). A formulation of the cooperative model for the yield stress of amorphous polymers for a wide range of strain rates and temperatures. Polymer.

[24] Jin T. (2016). Yield Behavior and Macro Phenomenological Constitutive Study of Semi-Crystalline Polymers. Ph.D.Thesis.

[25] Rao J., Xu W.L. (2009). Mechanical properties of UHMWPE fiber under heating state. J. Text..

[26] Bauwens-Crowet C., Bauwens J.C., Homes G. (1972). The temperature dependence of yield of polycarbonate in uniaxial compression and tensile tests. J. Mater. Sci..

[27] Baozhong S., Bohong G., Xin D. (2005). Compressive behavior of 3-D angle-interlock woven fabric composites at various strain rates. Polym. Test..

[28] Sun Y., Wang G.J., Zhang D.T., Chen L., Zhang M. (2011). Experimental investigation on dynamic compression properties of UHMWPE/Vinyl ester 2.5 dimensional angle interlocked woven composites. J. Mater. Eng..

[29] Wang L.L. (2005). Stress Wave Foundation.

[30] Malvern L.E. (1951). Plastic wave propagation in a bar of material exhibiting a strain rate effect. Q. Appl. Math..

[31] Frost C.C. (1992). Constitutive modeling and simulation of energy absorbing polyurethane foam under impact loading. Polym. Eng. Sci..

[32] Mu L.J. (2017). Study on Strain Rate Dependent Constitutive Model of Typical Polymer Materials. Master’s Thesis.

[33] Nagy A., Ko W.L., Lindholm U.S. (1974). Mechanical behavior of foamed materials under dynamic compression. J. Cell. Plast..

[34] Xu L.Z., Gao G.F., Zhao Z., Wang J.B., Cheng C. (2019). Compressive mechanical properties of polyethylene at different strain rates. Explos. Shock. Waves.

